# Agreement between self-reported and measured weight and height collected in general practice patients: a prospective study

**DOI:** 10.1186/1471-2288-13-38

**Published:** 2013-03-13

**Authors:** Sze Lin Yoong, Mariko Leanne Carey, Catherine D’Este, Robert William Sanson-Fisher

**Affiliations:** 1 Priority Research Center for Health Behavior & Hunter Medical Research Institute, Newcastle Australia, University of Newcastle, Callaghan, 2308, Australia; 2 Center for Clinical Epidemiology and Biostatistics, University of Newcastle, Callaghan, 2308, Australia

**Keywords:** Obesity, Family practice, Weight

## Abstract

**Background:**

Self-reported weight and height is frequently used to quantify overweight and obesity. It is however, associated with limitations such as bias and poor agreement, which may be a result of social desirability or difficulties with recall. Methods to reduce these biases would improve the accuracy of assessment of overweight and obesity using patient self-report. The level of agreement between self-reported and measured weight and height has not been widely examined in general practice patients.

**Methods:**

Consenting patients, presenting for care within four hour sessions, were randomly allocated to the informed or uninformed group. Participants were notified either a) *prior* to (informed group), or b) *after* (uninformed group) reporting their weight and height using a touchscreen computer questionnaire, that they would be measured. The differences in accuracy of self-report between the groups were examined by comparing mean differences, intraclass correlations (ICCs), Bland Altman plot with limits of agreement (LOAs) and Cohen’s kappa. Overall agreement was assessed using similar statistical methods.

**Results:**

Of consenting participants, 32% were aged between 18–39 years, 42% between 40–64 years and 25% were 65 years and above. The informed group (n = 172) did not report their weight and height more accurately than the uninformed group (n = 160). Mean differences between self-reported and measured weight (p = 0.4004), height (p = 0.5342) and body mass index (BMI) (p = 0.4409) were not statistically different between the informed and uninformed group. Overall, there were small mean differences (−1.2 kg for weight, 0.8 for height and −0.6 kg/m^2^ for BMI) and high ICCs (>0.9) between self-reported and measured values. A substantially high kappa (0.70) was obtained when using self-reported weight and height relative to measured values to quantify the proportion underweight, normal weight, overweight or obese. While the average bias of self-reported weight and height as estimates of the measured quantities is small, the LOAs indicate that substantial discrepancies occur at the individual level.

**Conclusions:**

Informing patients that their weight and height would be measured did not improve accuracy of reporting. The use of self-reported weight and height for surveillance studies in this setting appears acceptable; however this measure needs to be interpreted with care when used for individual patients.

## Background

Overweight and obesity affects a large proportion of the population in the developed world [1]. As the access point for health care systems in countries including Australia, Canada and United Kingdom, general practice is a valuable setting to target the reduction of overweight or obesity. GPs have access to a large proportion of the population, with 81% of Australians aged 15 years and above reporting having consulted their GP at least once a year [2]. Both GPs [3] and patients [4,5] perceive weight management to be part of a physicians’ role and those who are advised to lose weight by a health care provider are more likely to attempt to lose weight [6].

Self-reported weight and height is commonly used to assess overweight and obesity as it enables the body mass index (BMI) to be calculated. In the general practice setting, self-reported weight and height is often utilised in large-scale monitoring studies, where it may not be feasible to carry out weight and height measurements. For example, the largest ongoing study with general practice patients in Australia (the Bettering the Evaluation and Care of Health (BEACH)) uses self-reported weight and height to provide surveillance data on prevalence of overweight or obesity [7]. While self-report is a relatively cost-effective, practical and less invasive way of obtaining weight and height, this method of assessment is subject to a number of limitations such as bias or poor agreement, which may be a result of social desirability or difficulties with recall [8]. Previous population studies have reported that using self-reported weight and height frequently leads to an underestimation of overweight and obesity when compared to measured values [9,10]. Most studies examining the accuracy of self-report have however been conducted in the general population [9,11-13]. In order to utilise self-report for monitoring of overweight or obesity in this setting, the accuracy of self-reported weight and height in general practice patients’ needs to be evaluated.

Simple strategies to improve self-reported weight and height could potentially be useful in helping improve surveillance of excess weight in general practice. One strategy that has been used to improve the reporting of socially undesirable behaviours is the bogus pipeline method [14]. Using this method, respondents are given the impression that the accuracy of their responses will be independently checked. It is underpinned by the assumption that people are more likely to tell the truth when they think that their responses will be verified [15]. Black et al. tested the effectiveness of a variation of this method in improving accuracy of self-reported weight and height in volunteers in a shopping mall [16]. Participants in the intervention group were informed that their weight and height would be measured and then asked to report their weight and height; whilst those in the control group reported their weight and height before being told that they would be measured [16]. Participant in the intervention group reported their weight and height significantly more accurately than those in the control group. Despite its’ potential to improve accuracy of self-reported weight and height, no other study examining this intervention exists, to our knowledge.

This study therefore aimed to test whether advising general practice patients that their height and weight would be measured was effective in improving accuracy of self-report. It also aims to provide an index of reliability and agreement for self-reported weight and height in general practice patients, collected using a touchscreen computer, using mean differences, intraclass correlations (ICC) and Bland Altman plots with 95% limit of agreements (LOAs). The impact of self-report on categorization of underweight, normal weight, overweight and obesity was also assessed using Cohen’s kappa. An additional aim was to determine whether mean difference in reporting of self-reported and measured weight, height and BMI varied by age category.

## Methods

This study was conducted as part of a larger study testing the acceptability of using a touchscreen computer questionnaire in twelve general practices in Australia [2]. A subsample of patients from three practices was invited to participate in the current study. Consecutive patients aged 18 years and above, presenting for an appointment to their GP and able to provide informed consent were eligible to participate. Patients were not excluded based on the presence of other health conditions. Research staff recorded the sex of all invited patients in order to assess for consent bias. Participants were randomised to the informed or uninformed group and completed a touchscreen computer questionnaire. Participants’ weight and height measurements were obtained after completion of the questionnaire.

### Experimental groups

General practice sessions (4 hours) were centrally randomised by the researcher to the informed or uninformed group using a random number table. Participants recruited within the one session were all allocated to the same group. Neither practice staff nor patients were aware of group allocation.

#### Informed group

Participants’ consent to have their weight and height measured was sought *prior* to commencement of the questionnaire. After consenting to have their measurements taken, participants provided their self-reported weight and height using the touchscreen questionnaire.

#### Uninformed group

Participants provided their self-reported height and weight as part of completion of the questionnaire. The research assistant asked for consent to obtain weight and height measurements *after* participants provided their self-reported weight and height.

### Variables

#### Self-report

Participants were asked to provide demographic information including gender and whether they had a government concession health card. Patients were asked to select their age from the following categories: 1 = 18-24; 2 = 25-29; 3 = 30-34; 4 = 35-39; 5 = 40-44; 6 = 45-49; 7 = 50-54; 8 = 55-59; 9 = 60-64; 10 = 65-69; 11 = 70 and above. Participants also reported weight in either kilograms (kg) or stones/pounds and height in centimetres (cm) or feet/inches. All weight responses were converted to kg and height response converted to cm.

#### Measured

Participants’ weight was obtained using a digital body fat and muscle weighing scale and height measured with participants head in the Frankfort plane using a mounted stadiometer. Participants were asked to remove their shoes, any heavy outer garments and personal belongings prior to measurement. Weight was measured to the nearest 0.1 kg and height to the nearest 0.1 cm. A trained anthropometrist took patients’ weight and height measurements twice. A third measurement was taken if there was more than a 10% variation between the first and second measurement.

### Ethical approval

Ethical approval was provided by the University of Newcastle Human Research Ethics Committee (H2009-0341) and ratified by the University of New South Wales HREC (HREC 09393/ UN H-2009-0341) and Monash University HREC (2009001860).

### Data analysis

STATA SE version 11.0 (StataCorp, College Station, Tex, USA) was used to perform all statistical analyses. Self-reported values of height larger than 240 cm and smaller than 120 cm and values of weight larger than 250 kg and less than 30 kg were coded as missing as these values were perceived to be errors in self-report. BMI was calculated from both self-reported and measured data using weight in kg divided by metres squared. Consent rates for physical measures were compared between the informed and uninformed groups. Differences between self-reported and measured values were obtained for weight, height and BMI. Mean differences, ICCs and corresponding 95% CIs for height, weight and BMI were tabulated separately for the informed and uninformed groups and compared between groups using student’s t-test for mean differences and by comparing 95% CIs for ICCs [17,18]. Bland Altman plots with 95% LOA for height, weight and BMI were generated for both groups. The Bland Altman test is a statistically robust method of assessing reliability and agreement [19]. Additionally, Cohen’s kappa statistic and 95% CI for classification of underweight (BMI <18.5 kg/m^2^), normal weight (BMI ≥ 18.5 kg/m^2^ and <25 kg/m^2^), overweight (BMI ≥25 kg/m^2^and <30 kg/m^2^) or obesity (BMI ≥ 30 kg/m^2^) was generated and compared between groups using 95% CIs. The overall level of agreement between self-reported and measured weight, height and BMI was also assessed. Mean differences between self-reported and measured values and corresponding standard deviations for males and females were reported. An ICC for the overall sample was calculated to provide an estimate of reliability. Cohen’s kappa was calculated to provide the level of agreement between self-reported and measured classification of BMI categories. The degree of agreement between patient measured and self-reported overweight and obesity was assessed as follows: κ < 0 is none/poor; 0 ≤ κ ≤ 0.20 is slight; 0.21 ≤ κ ≤ 0.40 is fair; 0.41 ≤ κ ≤ 0.60 is moderate; 0.61 ≤ κ ≤ 0.80 is substantial; and 0.81 ≤ κ ≤ 1.0 is almost perfect [20]. Mean differences in self-reported weight and height were reported by age group. An ANOVA test was carried out to compare the mean difference in reporting by age (collapsed as 18–24, 25–44, 45–64 and ≥65 years).

### Sample size calculation

An initial sample size calculation was calculated to detect a difference of 0.5 kg/m^2^ in mean BMI between the two groups, with 80% power and 95% significance level, assuming a standard deviation of 1.5. To achieve this, a minimum of 142 participants needed to be recruited into each group (284 patients overall). A sample of this size would allow detection of a difference +/− 0.02 in mean ICCs between groups with 80% power, at 5% significance, assuming a standard deviation of 0.5. For overall agreement, this number of patients would allow estimation of kappa with 95% confidence within +/− 0.1, for a kappa of 0.4 or higher [21]. This sample size would also allow us to detect an ICC of 0.7 or more as being significantly greater than 0.6 [22]. A sample size of approximately 300 (75 per age group) would have at least 80% power, with 5% significance, to detect a difference in the variation between self-reported and measured weight, height and BMI of 0.6 standard deviations.

## Results

Overall, 86% of patients consented to completing the questionnaire for the larger study. 355 patients were asked if they were willing to have their weight and height measured and 93% (*n* = 332) consented. No significant differences in proportion of males and females who consented to and did not consent to being measured were identified (χ^2^: 1.1304, df = 1; p = 0.288). There was no significant difference in the proportion who consented to being measured between the informed (93%) and uninformed (92%) group (χ^2^: 0.9213, df = 1; p = 0.337).

Eleven participants reported having a height of more than 240 cm or less than 120 cm and/or having a weight of more than 250 kg or less than 30 kg. One participant in the uninformed group was excluded as the height difference tabulated was beyond reasonable error rate. More than half (56%) of consenting participants were female, 25% were aged 65 years and above and 42% had a government subsidised health care card. 14.2% of the Australian population are aged 65 years above [23]. While not directly comparable due to the inclusion of those aged below 18 in the latter population statistics, the current sample had a larger proportion of older people (≥65 years) than would be expected in the general population. This larger proportion of older participants is consistent with that identified in other general practice datasets [7]. Demographic characteristics are presented for the informed (*n* = 172) and uninformed (*n* = 160) groups (see Table 1).

**Table 1:** Mean difference and intraclass correlation for weight, height and BMI for informed and uninformed group

**Group**	Informed (n = 172)*	Uninformed (n = 160)**
*Sex n (%)*		
Male	78 (45)	69 (43)
Female	94 (55)	91 (57)
*Age n (%)*		
18-39	60 (35)	48 (30)
40-64	67 (39)	74 (46)
65 +	45 (26)	38 (24)
*Number with concession health care (%)*	71 (42)	66 (42)
*Mean difference*^a^*(sd) [95% CI]*		
Weight	−1.0 (4.5) [−9.8, 7.8]	−1.4 (3.5) [−8.1,5.4]
Height	0.7 (3.9) [−6.9, 8.2]	1.0 (5.0) [−8.7, 10]
BMI	−0.6 (2.2) [−4.8, 3.7]	−0.7 (1.9) [−4.4,3.0]
*ICC [95% CI]*		
Weight	0.97 [0.96, 0.98]	0.98 [0.97, 0.99]
Height	0.92 [0.90, 0.94]	0.90 [0.87, 0.93]
BMI	0.93 [0.91, 0.95]	0.94 [0.93, 0.96]

ICC: Intraclass correlation; CI: confidence interval; BMI: Body Mass Index; sd: standard deviation.

^a^ mean difference calculated using self-reported weight minus measured weight.

*Missing data for informed group: 3 missing for weight, 3 missing for height; subsequently 6 missing for BMI.

**Missing data for uninformed group: 2 missing for weight, 6 missing for height; subsequently 6 missing for BMI.

There were no significant differences in mean difference of self-reported and measured weight (p = 0.4004), height (p = 0.5342) and BMI (p = 0.4409) and ICCs between the informed and uninformed group (see Table 1).

When measured and self-reported BMI categories were examined the percentage agreement was 78% for the informed group and 81% for the uninformed group. The kappa values were 0.68 [95% CI 0.58, 0.78] for the informed and 0.72 [95% CI 0.61, 0.83] for the uninformed group and overlap between 95% CIs indicated no significant differences.

**Figure 1:**
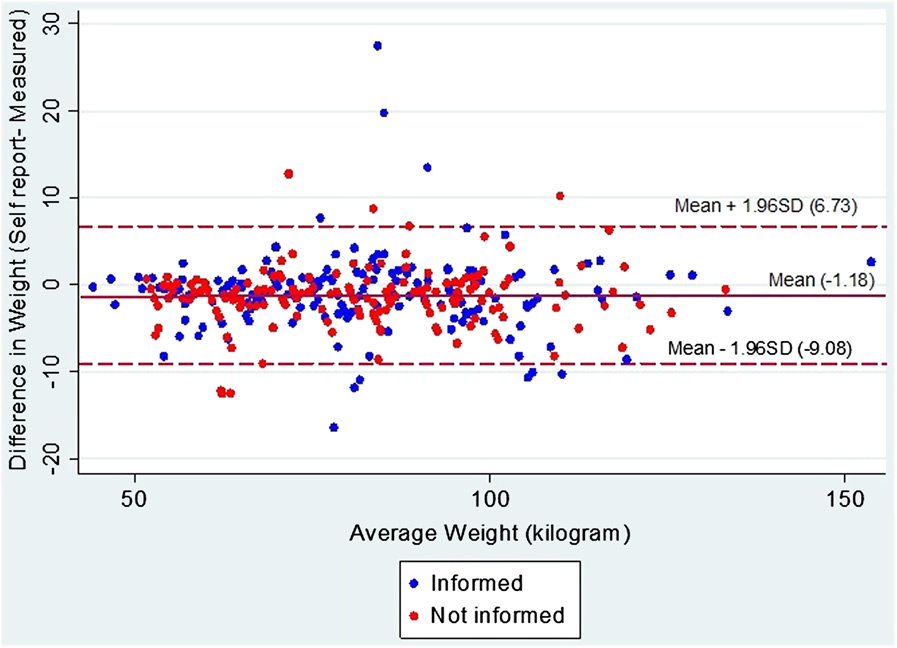
**Bland Altman plot for self-reported and measured weight (in kilograms) in informed and uniformed general practice patients.** Middle line represents mean difference of methods. Lines above and below represent 95% limits of agreements (LOA), where upper LOA is +1.96 SD and lower line is −1.96 SD from overall mean differences.

**Figure 2:**
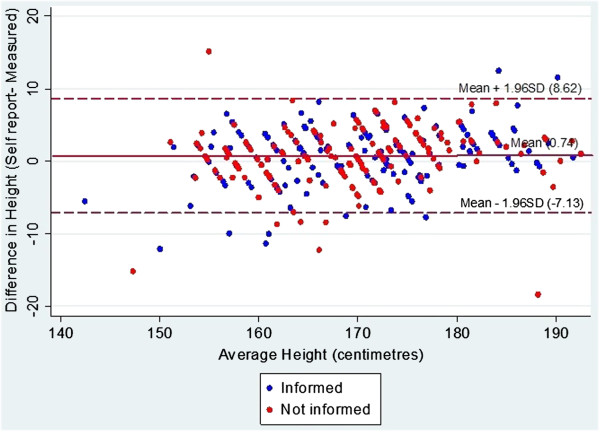
**Bland Altman plot for self-reported and measured height (in centimetres) in informed and uniformed general practice patients.** Middle line represents mean difference of methods. Lines above and below represent 95% limits of agreements (LOA), where upper LOA is +1.96 SD and lower line is −1.96 SD from overall mean differences.

**Figure 3:**
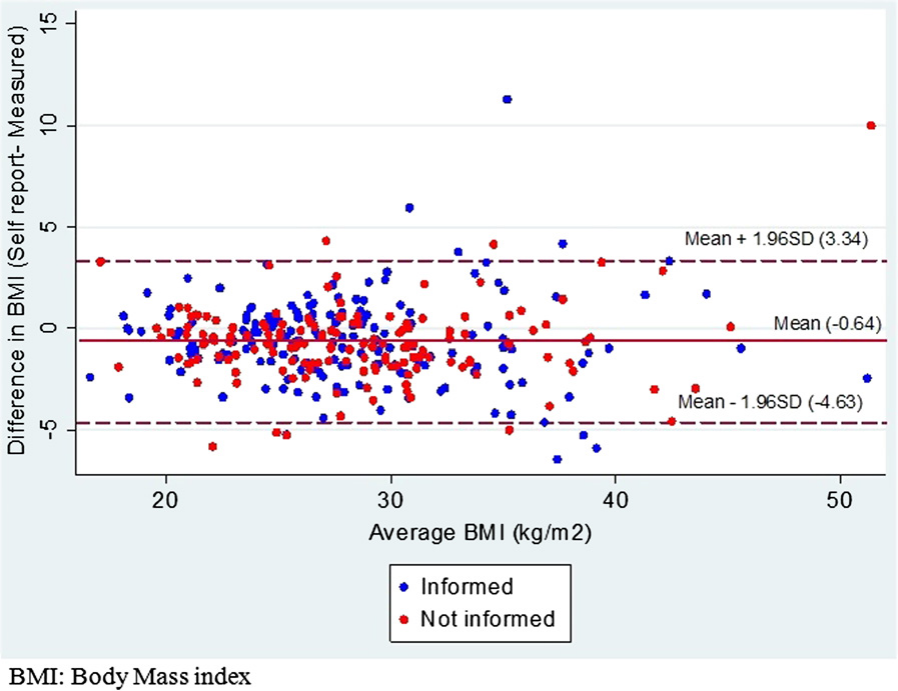
**Bland Altman plot for self-reported and measured body mass index (in kg/m**^**2**^**) in informed and uniformed general practice patients.** Middle line represents mean difference of methods. Lines above and below represent 95% limits of agreements (LOA), where upper LOA is +1.96 SD and lower line is −1.96 SD from overall mean differences.

The Bland-Altman plots for weight, height and BMI for the informed and uninformed groups are shown in Figures 1, 2 and 3.

As there were no significant differences in accuracy of self-reported weight and height between the groups, the sample was pooled to assess overall agreement and reliability. Overall mean differences between self-reported and measured values were −1.2 kg (4.0) for weight [males −1.2 kg (3.6), females −1.2 kg (4.4)], 0.8 cm (4.4) for height [males 1.5 cm (3.5); females 0.3 cm (4.9)] and −0.6 kg/m^2^ (2.0) for BMI [males −0.9 kg/m^2^ (1.7); females −0.4 kg/m^2^ (2.3)]. The overall ICCs for self-reported and measured values and their corresponding 95% CIs were 0.97 [0.97, 0.98] for weight, 0.91 [0.89, 0.93] for height and 0.94 [0.91, 0.95] for BMI. The Bland Altman plots and LOA provide an indication of the extent of underreporting and overreporting of weight, height and BMI when compared to measured values (see Figures 1, 2, 3).

The overall percentage agreement between self-reported and measured classification of BMI categories was 80% [95% CI 75, 84]. Twenty percent of those who were overweight were categorised as normal weight using self-reported weight and height (see Table 2). Of those who were obese, 22% were classified as overweight using self-reported weight and height. The prevalence of obesity was underestimated by 5% (35% using measured and 30% using self-report) and prevalence of normal weight was overestimated by 5% (27% using measured and 32% using self-report). The kappa for categorisation of BMI was 0.70 [95% CI 0.63, 0.77]; representing substantial agreement [20] and that level of agreement was greater than expected by chance alone (p < 0.001).

**Table 2:** Categorisation of body mass index (BMI) category based on self-reported and measured weight and height

	**Measured n (%)**^**b**^				**Total**^**a**^
**Self-report**	**Underweight**	**Normal weight**	**Overweight**	**Obese**	
Underweight	4 (67)	2 (2.3)	0 (0)	0 (0)	6 (1.9)
Normal weight	2 (33)	78 (91)	23 (20)	0 (0)	103 (32)
Overweight	0 (0)	6 (7.0)	85 (74)	25 (22)	116 (36)
Obese	0 (0)	0 (0)	7 (6.1)	88 (78)	95 (30)
**Total**	6 (1.9)	86 (27)	115 (36)	113 (35)	320

^a^ Total less than overall included due to invalid values in self-reported weight and height.

^b^ Percentage reported is proportion of measured for each BMI category.

Underweight defined as BMI <18.5 kg/m^2^; Normal weight defined as BMI ≥18.5 kg/m^2^ and <25 kg/m^2^; Overweight defined as BMI ≥25 kg/m^2^ and <30 kg/m^2^; Obese defined as BMI ≥30 kg/m^2^.

There were no significant differences by age, when mean differences in measured and self-reported weight, height and BMI were compared (see Table 3).

**Table 3:** Mean difference between measured and self-reported weight, height and BMI by age categories in Australian general practice patients

	Mean difference* (standard deviation)				**ANOVA test**		
	**18 – 24**	**25 – 44**	**45 – 64**	≥**65**	**F**	**df**	**p-value**
	**(*n*** **= 33)**	**(*n =*** **95)**	**(*n =*** **121)**	**(*n =*** **83)**			
Weight	−1.6 (0.6)	−0.8 (0.4)	−1.1 (0.4)	−1.6 (0.4)	0.73	3	0.5344
Height	−0.004 (1.0)	1.2 (0.6)	0.7 (0.3)	0.9 (0.4)	0.67	3	0.5695
BMI	−0.6 (0.3)	−0.5 (2.0)	−0.6 (0.2)	−0.9 (0.2)	0.63	3	0.5950

*Mean calculated using measured self-reported minus measured values.

## Discussion

This study demonstrated that informing general practice patients that their height and weight would be measured did not improve accuracy of self-report. This contrasts with Black and colleagues’ findings, where those who were informed that they would be measured reported their weight and height significantly more accurate than those who were not informed [16]. This difference in findings could have occurred due to several differences in study methodology, setting, participants and statistical analyses conducted. Black and colleagues recruited their sample for a health screen in a shopping mall whereas the current study recruited participants presenting for general practice care. General practice patients may be more willing to disclose their weight and height compared to volunteers in a shopping mall. Further, Black’s study included only participants aged between 18 to 28 years whereas only a small proportion (18%) of our sample was aged between 18 and 29 years [16]. Those in the younger age bracket may be more likely to be affected by cultural ideals regarding weight and height [24], which might have led to attempts to misreport these measures. Inconsistent findings regarding the accuracy of self-reported weight and height in older patients have been identified, with one longitudinal study reporting that only small changes in reporting of weight and height occurred with increasing age [25] and others identifying substantial differences between measured and self-reported weight and height in those older [26,27]. Our study did not find any significant differences in mean reporting of self-reported and measured values with age category; and no pattern of increasing or decreasing difference with age was observed. Black and colleagues also asked participants in the informed group six additional ‘body weighing questions’ which may have helped with recall of weight [16]. The current study aimed to test solely if informing patients that their weight and height would be measured would improve accuracy of self-report and thus did not include these questions. Given that GPs see a larger proportion of older patients, reporting of weight and height in this group may be less affected by social desirability bias and suggests that misreporting may be attributed largely to recall bias or not having an accurate knowledge of one’s current weight and height. Future studies testing this intervention in younger patients may produce different findings.

Consistent with findings in other populations [8], participants in the present study tended to underreport their weight and overreport their height. Mean differences between self-reported and measured weight (−1.2 kg in males; -1.2 kg in females) and height (1.5 cm in males; 0.3 cm in females) are within the range of that identified in a review examining accuracy of self-reported weight (−1.9 kg to 0.4 kg in males; -1.6 kg to 0.7 kg in females) and height (−1.3 cm to 2.3 cm in males; -1.7 cm to 2.2 cm in females) in the general population [8]. Only one Australian study was included in the review, however this study did not report mean differences. When compared to other Australian studies, our study had lower mean differences in self-reported and measured values for reporting of height and weight, particularly for females. Taylor et al. identified mean differences of −1.5 kg in males; -1.8 kg in females for weight and 1.4 cm in males; 1.3 cm in females for height [12]. Another study identified mean differences of 2.0 cm in males; 0.8 cm in females for height and −1.4 kg for males and −3.0 kg for females [13]. There is some evidence to suggest that females who had recently consulted a doctor may be able to more accurately recall their weight and height [28]. Additionally, patients presenting for care to their GP may represent a more ‘health conscious’ sample and thus, may be more aware of their weight and height measurements. Differences could also be attributed to the fact there was no time lag between self-report and measured assessments in our study, whereas an average of 23.5 days between self-reported and measured data was reported in the study conducted by Taylor [12].

Overall, 80% of participants were accurately classified as underweight, normal weight, overweight or obese using self-reported weight and height. The use of self-reported BMI resulted in no difference in prevalence of overweight and only a 5% lower prevalence of obesity when compared to estimates obtained using measured data. These findings are favourable when compared to other studies which indicate that self-reported data underestimated the proportion of participants classified as overweight by 2% to 12% and obese by approximately 7% [12,13]. The present findings however, are comparable to the 2008 Australian National Health Survey, which identified a 6% rate of underestimation of prevalence of overweight or obese when self-reported data was compared to measured data [10].

While the current study identified high reliability between self-reported and measured weight and height, represented by high ICCs (>0.9) for weight, height and BMI, the estimated Bland Altman LOAs suggests that accuracy of individuals’ self-report may vary. When compared to measured weight, inaccuracies in self-reported weight ranged from overestimation of 6.7 kg to underestimation of 9.1 kg. Similarly, inaccuracies in self-reported height ranged from an overestimation of 8.6 cm to underestimation of 7.1 cm. This subsequently led to overreporting of BMI by 3.3 kg/m^2^ and underreporting of up to 4.6 kg/m^2^.

### Strengths and limitations

A high consent rate was achieved, with 93% agreeing to have their weight and height measured. This high consent rate may be due to the use of the touchscreen computer which could have provided participants with a more private way of reporting weight and height. There was no time lapse between provision of self-report and actual measurement of weight and height, thus reducing potential error attributed to weight change during the time lapse. The use of ICCs and Bland Altman plots with LOAs provide a more robust examination of agreement compared to the more traditionally used Pearson’s correlation coefficients as it provides an indication of variability and agreement rather than association. The ICC however treats self-reported and measured values as exchangeable (i.e. method of measurement is assumed to be a random effect). When systematic differences between methods of measurement occur, high ICCs may not necessarily imply high agreement.

Some of the variation between self-reported values and measured values may be accounted for by the way in which participants report their weight and height (e.g. end-digit preferences [12] and reporting in imperial units rather than metric). A large proportion of participants included in this study were aged 65 years and above. However, when mean differences in self-reported and measured values were compared, no differences were identified between older and younger participants. This study was conducted in only three practices, potentially limiting the generalizability of findings. No significant differences in participant’s sex was observed when compared to a larger Australian general practice dataset (BEACH), which included 95,839 patient encounters recruited by 958 GPs [7]. However, a difference in distribution of age was observed between our sample and the BEACH dataset [7].

## Conclusion

Informing general practice patients that their weight and height would be measured did not significantly improve accuracy of self-report. Testing this strategy in subgroups likely to be affected by cultural ideals regarding weight (i.e. younger, female) may be beneficial in helping identify ways to improve accuracy of self-report for these groups. Self-reported weight and height provides relatively accurate estimates of BMI in Australian general practice patients. Thus, in circumstances where population trends are of interest such as in large surveillance studies, self-report is likely to be an accurate alternative. While the average bias of self-reported weight and height as estimates of the measured quantities is small, the LOAs indicate that there is a need for these values to be interpreted with caution in individuals.

## Competing interest

The authors declare no competing interest.

## Authors’ contributions

SY, MC, CD and RSF all participated in conception of the study and survey design. SY conducted all data collection and initial data analysis. SY, MC and CD had input into the statistical analysis. All authors offered critical comments on the draft of the manuscript and approved the final submitted version.

## Pre-publication history

The pre-publication history for this paper can be accessed here:

http://www.biomedcentral.com/1471-2288/13/38/prepub

## References

[B1] World Health Organization (WHO) Obesity and overweight Available from: http://www.who.int/mediacentre/factsheets/fs311/en/

[B2] Australian Bureau of Statistics Health Service: Use and patient experience *Available from:* http://www.abs.gov.au/ausstats/abs@.nsf/Lookup/BF1313C0400DA15BCA25792E000D5B86?opendocument

[B3] Campbell K Engel H Timperio A Cooper C Crawford D Obesity management: Australian General Practitioners’ Attitudes and Practices Obes Res 2000 8 6 459 466 10.1038/oby.2000.57 11011913

[B4] Tham M Young D The role of the General Practitioner in weight management in primary care–a cross sectional study in General Practice BMC Fam Pract 2008 9 66 10.1186/1471-2296-9-66 19077319 PMC2614998

[B5] Tan D Zwar NA Dennis SM Vagholkar S Weight management in general practice: what to patients want? Med J Aust 2006 185 2 73 75 16842059 10.5694/j.1326-5377.2006.tb00474.x

[B6] Galuska DA Will JC Serdula MK Ford ES Are health care professionals advising obese patients to lose weight? JAMA 1999 282 16 1576 1578 10.1001/jama.282.16.1576 10546698

[B7] Britt H Miller GC Charles J Henderson J Bayram C Valenti L Harrison C Pan Y O’Halloran J Zhang C General practice activity in Australia 2010–11. General practice series no. 29 2011 Sydney: Sydney University Press

[B8] Gorber SC Tremblay M Moher D Gorber B A comparison of direct vs. self-report measures for assessing height, weight and body mass index: a systematic review Obes Rev 2007 8 4 307 326 10.1111/j.1467-789X.2007.00347.x 17578381

[B9] Elgar FJ Stewart JM Validity of self-report screening for overweight and obesity. Evidence from the Canadian Community Health Survey Can J Public Health 2008 99 5 423 427 19009930 10.1007/BF03405254 PMC6975656

[B10] Australian Bureau of Statistics 4364.0- National Health Survey: Summary of Results, 2007–2008 (Reissue) Available online from: http://www.abs.gov.au/AUSSTATS/abs@.nsf/Lookup/4364.0Main+Features12007-2008%20%28Reissue%29?OpenDocument

[B11] Burton NW Brown W Dobson A Accuracy of body mass index estimated from self-reported height and weight in mid-aged Australian women Aust NZ J Publ Heal 2010 34 6 620 623 10.1111/j.1753-6405.2010.00618.x 21134066

[B12] Taylor AW Grande ED Gill TK Chittleborough CR Wilson DH Adams RJ Grant JF Phillips P Appleton S Ruffin RE How valid are self-reported height and weight? A comparison between CATI self-report and clinic measurements using a large cohort study Aust N Z J Public Health 2006 30 3 238 246 10.1111/j.1467-842X.2006.tb00864.x 16800200

[B13] Flood V Webb K Lazarus R Pang G Use of self-report to monitor overweight and obesity in populations: some issues for consideration Aust N Z J Public Health 2000 24 1 96 99 10.1111/j.1467-842X.2000.tb00733.x 10777989

[B14] Roese NJ Jamieson DW Twenty years of bogus pipeline research: a critical review and meta-analysis Psychol Bull 1993 114 2 363 375

[B15] Jones EE Sigall H The bogus pipeline: a new paradigm for measuring affect and attitude Psychol Bull 1971 76 5 349 364

[B16] Black DR Taylor AM Coster DC Accuracy of self-reported body weight: Stepped Approach Model component assessment Health Educ Res 1998 13 2 301 307 10.1093/her/13.2.301 10181028

[B17] Shrout P Fleiss J Intraclass correlations: uses in assessing rater reliability Psychol Bull 1979 86 2 420 428 18839484 10.1037//0033-2909.86.2.420

[B18] Morton AP Dobson AJ Assessing agreement Med J Australia 1989 150 7 384 387 2716662 10.5694/j.1326-5377.1989.tb136531.x

[B19] Bland JM Altman DG Statistical methods for assessing agreement between two methods of clinical measurement Lancet 1986 1 8476 307 310 2868172

[B20] Landis JR Koch GG The measurement of observer agreement for categorical data Biometrics 1977 33 1 159 174 10.2307/2529310 843571

[B21] Flack VF Afifi AA Lachenbruch PA Schouten HJA Sample size determinations for the two rater kappa statistic Psychometrika 1988 53 3 321 325 10.1007/BF02294215

[B22] Shoukri MM Asyali MH Donner A Sample size requirements for the design of reliability study: review and new results Stat Methods Med Res 2004 13 4 251 271

[B23] Australian Bureau of Statistics Australian Demographic Statistics *Available from:* http://www.abs.gov.au/ausstats/abs@.nsf/mf/3101.0

[B24] Ziebland S Thorogood M Fuller A Muir J Desire for the body normal: body image and discrepancies between self reported and measured height and weight in a British population J Epidemiol Community Health 1996 50 1 105 106 10.1136/jech.50.1.105 8762365 PMC1060215

[B25] Dahl AK Hassing LB Fransson EI Pedersen NL Agreement between self-reported and measured height, weight and body mass index in old age–a longitudinal study with 20 years of follow-up Age Ageing 2010 39 4 445 451 10.1093/ageing/afq038 20453247 PMC2899942

[B26] Lawlor DA Bedford C Taylor M Ebrahim S Agreement between measured and self‐reported weight in older women. Results from the British Women’s Heart and Health Study Age Ageing 2002 31 3 169 174 10.1093/ageing/31.3.169 12006304

[B27] Kuczmarski MF Kuczmarski RJ Najjar M Effects of age on validity of self-reported height, weight, and body mass index: findings from the Third National Health and Nutrition Examination Survey, 1988–1994 J Am Diet Assoc 2001 101 1 28 34 quiz 35–26 10.1016/S0002-8223(01)00008-6 11209581

[B28] Villanueva E The validity of self-reported weight in US adults: a population based cross-sectional study BMC Public Health 2001 1 1 11 10.1186/1471-2458-1-11 11716792 PMC59896

